# Skin Necrosis Associated with Thromboprophylaxis after Total Knee Replacement

**DOI:** 10.1155/2014/139218

**Published:** 2014-04-06

**Authors:** S. V. Karuppiah, A. J. Johnstone

**Affiliations:** ^1^Department of Trauma & Orthopaedics, Grampian University Hospitals NHS Trust, Aberdeen Royal Infirmary, Aberdeen AB25 2ZN, UK; ^2^4, Drake Close, Hethersette, Norwich NR9 3JS, UK

## Abstract

Thromboprophylaxis are routinely given to prevent venous thromboembolism (VTE) in patients after total hip and knee replacement surgeries. Low molecular weight heparin (LMWH) (fractioned heparin) is effective in the prevention and treatment of VTE. The predicable effect of LMWH has popularized it for routine clinical use. Although LMWH has lesser complication rate, compared to unfractioned heparin (UFH), sporadic clinical complication has been reported. We report a rare case of skin necrosis secondary to use of LMWH tinzaparin used for routine thromboprophylaxis after total knee replacement.

## 1. Introduction

Low molecular weight heparin (LMWH) was developed in the late 1970s after the elucidation of heparin's structure and the identification of the pentasaccharide as being its minimal active fragment. LMWHs have proven to be well tolerated and effective in the prevention and treatment of venous thromboembolism (VTE). The advantages of LMWHs include its predictable anticoagulant response, an improved bioavailability, and a longer half-life [1] which have replaced the traditional use of unfractioned heparin (UFH) to prevent VTE in patients undergoing total hip and knee replacement surgeries [2, 3].

Adverse effects of LMWHs are also quite uncommon making it advantageous compared to unfractioned heparin, probably because of the smaller size of the molecules, the greater homogeneity of the substance, and the exclusive porcine origin of the new compounds.

Skin reactions, a recognised complication of heparin products, have been reported with the use of UFH. However, there are only a few reports of LMWH causing skin reactions. We report a rare case of skin necrosis secondary to the use of LMWH Tinzaparin.

## 2. Case Report

A 67-year-old female patient, of BMI 30, suffering from severe osteoarthritis of her knee, underwent an elective total knee replacement surgery. She did not have any significant past medical history and had a normal blood profile prior to surgery. There were no intraoperative complications or immediate postoperative complications. As a routine postoperative care, she was administered prophylactic subcutaneous tinzaparin (low molecular weight heparin) 3,500 IU once a day on her abdomen.

**Figure 1:**
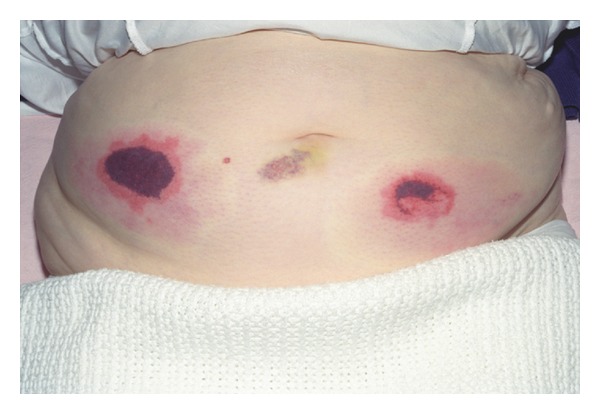
Skin necrosis on anterior abdominal wall after treatment with Tinzaparin.

On postoperative day 14, she developed an area of erythema associated with pain at the sites of her LMWH injection (Figure 1). Blood investigation revealed a normal platelet count of 254 (normal range 143–332 × 10^9^) with Haemoglobin of 12.5 (normal range 3.9–15 × 10^9^) and WCC 10.5 (normal range 3.9–11.1 × 10^9^).

Dermatology consultation was taken and vasculitis secondary to localized heparin-induced thrombocytopenia (HIT) was diagnosed. Repeat blood investigations did not show any change in the platelet count. With subsequent days, her abdomen skin broke down, and she developed an ulcer.

Tinzaparin was stopped and an alternative thromboprophylaxis, Aspirin 150 mg once a day, was started. She made good progress from her knee replacement and was subsequently discharged.

At a 6-week routine outpatient clinic follow-up, the patient had a good range of knee movement. At that time her abdomen wounds were healing with regular alternative day dressing. At 3-month follow-up, her wounds were completely healed.

## 3. Discussion

Adverse effects are less frequent with LMWHs than with unfractioned heparin (UFH) [4, 5]. The major adverse effects of UFH such as bleeding, thrombocytopenia, osteoporosis, hypoaldosteronism, alopecia, and skin reactions are not common with LMWHs [4, 5].

However, skin reactions, as seen in this patient, may rarely occur. Not many reports have been published and the mechanism is poorly understood. Skin reactions to heparin were first described by Plancherel and their frequency is estimated to be less than 0.2% [6–8]. Severe complications such as skin necrosis are certainly very rare; Fried et al. reported that enoxaparin sodium, one of the first marketed LMWHs, observed 2 cases of skin necrosis [9].

A review of literature showed that the skin reaction can be (i) as an urticarial rash, presumably due to local histamine release, (ii) as a classic type I immediate hypersensitivity reaction, (iii) as skin necrosis, often caused by vasculitis (type III Arthus reaction) or heparin-induced thrombocytopenia, or (iv) as a type IV delayed hypersensitivity reaction [10]. The clinical presentation in this patient shows it is of type III reaction.

Several pathophysiological mechanisms have been proposed [11], vasculitis induced by type III hypersensitivity reaction (Arthus phenomenon with deposit of immune complexes on endothelial structures) is most likely the cause in this patient. Other proposed theories are; (i) local trauma at injection sites; (ii) heparin-induced thrombocytopenia, which is triggered by the binding of antibodies to a platelet-heparin complex, leading to platelet activation and aggregation; (iii) poor vascularisation of adipose tissue inducing a diminished absorption of heparin, as seen in diabetic lipodystrophy [12].

Adverse skin reactions to low molecular weight heparins (LMWH) are rare even though their true incidence is probably underestimated because of underreporting. These reactions may occur as an urticarial rash, presumably due to local histamine release or have the features of a classic type I immediate hypersensitivity reaction. They can also present as skin necrosis often due to vasculitis (type III Arthus reaction) or heparin-induced thrombocytopenia. Erythematous, well circumscribed lesions without necrosis, are usually secondary to a delayed type IV hypersensitivity reaction.

Clinically, the lesions are usually located at injection sites, with well circumscribed, erythematous infiltrated plaques, often accompanied by pruritus. However skin necrosis has also been observed at locations distant from the site of subcutaneous heparin injection, or after intravenous heparin therapy [13, 14]. The lesions may rapidly become hemorrhagic and necrotic which can result in deep skin necrosis. These cutaneous lesions have also been reported in the use of warfarin-induced skin necrosis, although these lesions differ from each other from a pathological point of view [15]. The major systemic complications related to skin necrosis are similar to those found in heparin induced thrombocytopenia, with multiple venous and arterial thromboses which can lead to a fatal outcome [16]. In the absence of thrombocytopenia, only few distant organ complications such as glomerulonephritis have been described [17].

Although LMWH are used quite commonly as routine prophylaxis in total hip and knee replacement surgery, it is not without its complications. LMWH-induced skin lesions are not preventable, but complications can be reduced with early recognisation and treatment should be discontinued, replaced by an alternative medication such as danaparoid sodium or hirudin. As in this case, localised vasculitis does not alter the platelet count and has very little prognostic value. In a type IV delayed hypersensitivity reaction, in the absence of severe, extensive, life-threatening mucocutaneous manifestations, a first-line pragmatic approach consists, in our view, of replacing the particular LMWH with another one.

In doubtful cases, subcutaneous provocation test, the compound can be used to clarify the risk. If all types of LMWH and danaparoid sodium are positive in skin testing, mechanical prevention or oral anticoagulants should be used, and intravenous injections of any kind of heparin should be avoided because of the potential risk of anaphylactic shock. In these patients, oral anticoagulation should be preferred, whenever possible. In conclusion, though rare, skin reactions to LMWH, particularly in middle-aged, obese women and during pregnancy, have important consequences which can be reduced by rapid diagnosis and appropriate management.
